# Relative Contribution of Gestational Weight Gain, Gestational Diabetes, and Maternal Obesity to Neonatal Fat Mass

**DOI:** 10.3390/nu12113434

**Published:** 2020-11-09

**Authors:** Delphine Mitanchez, Sophie Jacqueminet, Said Lebbah, Marc Dommergues, David Hajage, Cécile Ciangura

**Affiliations:** 1 Department of Neonatology, Bretonneau Hospital, François Rabelais University, 37000 Tours, France; 2 INSERM, UMR_S 938 Saint Antoine Research Centre, Sorbonne University, 75012 Paris, France; 3 Department of Diabetology, Institute of Cardiometabolism And Nutrition (ICAN), Pitié Salpêtrière Hospital, Assistance Publique-Hôpitaux de Paris (APHP), Sorbonne University, 75013 Paris, France; sophie.jacqueminet@aphp.fr (S.J.); cecile.ciangura@aphp.fr (C.C.); 4 Clinic Research Unit, Pitié Salpêtrière Hospital, Assistance Publique-Hôpitaux de Paris (APHP), Sorbonne University, 75013 Paris, France; said.lebbah@aphp.fr; 5 Department of Gynaecology and Obstetrics, Pitié Salpêtrière Hospital, Assistance Publique-Hôpitaux de Paris (APHP), Sorbonne University, 75013 Paris, France; marc.dommergues@aphp.fr; 6 INSERM, Public Health Department, Pierre Louis Institute of Epidemiology and Public Health, AP-HP, Centre of Pharmacoepidémiology (Cephepi), Sorbonne University, 75013 Paris, France; david.hajage@aphp.fr; 7 Department of Nutrition, Institute of Cardiometbolism And Nutrition (ICAN), Pitié Salpêtrière Hospital, Assistance Publique-Hôpitaux de Paris (APHP), Sorbonne University, 75013 Paris, France

**Keywords:** neonatal fat mass, leptin, birthweight, obesity, diabetes

## Abstract

Maternal nutritional and metabolic status influence fetal growth. This study investigated the contribution of gestational weight gain (GWG), gestational diabetes (GDM), and maternal obesity to birthweight and newborn body fat. It is a secondary analysis of a prospective study including 204 women with a pregestational body mass index (BMI) of 18.5–24.9 kg/m^2^ and 219 women with BMI ≥ 30 kg/m^2^. GDM was screened in the second and third trimester and was treated by dietary intervention, and insulin if required. Maternal obesity had the greatest effect on skinfolds (+1.4 mm) and cord leptin (+3.5 ng/mL), but no effect on birthweight. GWG was associated with increased birthweight and skinfolds thickness, independently from GDM and maternal obesity. There was an interaction between third trimester weight gain and GDM on birthweight and cord leptin, but not with maternal obesity. On average, +1 kg in third trimester was associated with +13 g in birthweight and with +0.64 ng/mL in cord leptin, and a further 32 g and 0.89 ng/mL increase in diabetic mothers, respectively. Maternal obesity is the main contributor to neonatal body fat. There is an independent association between third trimester weight gain, birthweight, and neonatal body fat, enhanced by GDM despite intensive treatment.

## 1. Introduction

During pregnancy, maternal obesity modifies fetal growth, and increases the risk of having a large for gestational age (LGA) baby [1]. However, maternal obesity is frequently associated with gestational diabetes and excessive gestational weight gain (GWG), greater than that recommended by the Institute of Medicine (IOM) guidelines. Maternal diabetes and high GWG independently modify fetal growth [2,3].

In these situations, excess in fetal growth is mainly due to an increase in body fat [4]. While genetic factors have a stronger relationship with fetal fat free mass, fetal fat mass development is mainly influenced by in utero environment [5]. Maternal metabolic and nutritional status modify the intrauterine environment. Indeed, maternal diabetes, maternal obesity, and increased GWG are now recognized as modifiable factors that increase fatness at birth and may act synergistically [6,7,8]. An increase in maternal lipids, triglycerides, and fatty acids, appears to be the predominant substrates in driving fetal macrosomia, although the combined effect in all fuels likely contributes to the increased fatness [9]. These changes in fetal growth and body composition have important implications for future metabolic and cardiovascular health [4].

We previously conducted a prospective exposure-matched cohort study to determine the relative contribution of maternal obesity and gestational diabetes on neonatal birthweight and fat mass, estimated by skinfold sum and cord serum leptin [10]. For this, we compared neonates born to normal weight women (body mass index, BMI, 18.5–24.9 kg/m^2^), to neonates born to obese women (BMI ≥ 30 kg/m^2^). In order to minimize the potential effect of maternal hyperglycemia on neonatal anthropometrics, screening, and subsequent treatment for gestational diabetes were enhanced. We showed that regardless of gestational diabetes, maternal obesity was not associated with increased birthweight, but it was associated with higher fat mass and leptin in girls, but not in boys.

In an attempt to further understand the pathophysiology of this increase in neonatal fat mass, we aimed to analyze the relative contribution of GWG, maternal obesity, and gestational diabetes in proxies of neonatal fat mass, i.e., skinfolds and cord leptin. We considered both total and trimester specific GWG. In addition, we constructed a specific model to explore if the effect of GWG on neonatal fat mass differed according to pre-gestational BMI, gestational diabetes, and neonatal sex.

## 2. Materials and Methods

This is a secondary analysis of data collected for the MOBENN study (clinical trial registration number: NCT02681588, ClinicalTrials.gov) [10]. The study received the approval of the Ile-de-France ethics committee on November 18, 2009 (CPP: Committee of Protection of the People—n 79–09). Briefly, inclusion criteria were pre-gestational BMI ≥ 30 kg/m^2^ (obese mothers) or BMI 18.5–24.9 kg/m^2^ (normal weight mothers), maternal age 18 years or greater and below 41 years, and singleton pregnancy. Exclusion criteria were initiation of antenatal care after 18 weeks, known type 1 or type 2 diabetes, obesity due to a genetic disorder or secondary to intracranial tumor or radiotherapy, bariatric surgery, chronic diseases other than obesity, and non-fluency in French. Only pregnancies resulting in a term livebirth were taken into account for analysis. Obese and normal weight women were matched 1:1 based on the following criteria: age ± 5 years, nulliparity or multiparity, gestational age at inclusion ± 4 weeks.

### 2.1. Maternal Weight Gain

Women were enrolled in the first trimester and had a monthly follow-up throughout pregnancy, such as general care offers in our country. Weigh was recorded at each monthly prenatal visit. Weight was measured in hospital clinics at the first prenatal visit and then monthly, on one scale that was regularly calibrated. Body weight was measured to the nearest 0.1 kg with subjects in indoor clothing and no shoes. Height was measured to the nearest 0.5 cm with a wall-mounted stadiometer, in the same conditions. BMI was calculated as weight divided by height squared.

Total pregnancy weight gain was calculated based on the difference between the last recorded weight within two weeks before delivery and the self-reported pre-pregnancy weight.

Relative weight gain was defined as the percentage of weight gain during pregnancy relative to pre-gestational weight. Total GWG was classified according to the guidelines issued by the Institute of Medicine (IOM) [11].

First trimester weight gain was defined as the difference between weigh recorded at 14 ± 2 weeks of gestation and the self-reported pre-pregnancy weight. Second trimester weight gain was defined as the difference between weight recorded at 28 ± 2 weeks of gestation and weight recorded at 14 ± 2 weeks of gestation. Third trimester weight gain was the difference between the last recorded weight before delivery and weight recorded at 28 ± 2 weeks of gestation.

All patients received the general pregnancy dietary recommendations according to the French national program of nutrition and health recommendations. Weight gain targets were explained but no specific intervention for weight management during pregnancy was proposed. Recommended weight gain was 11 to 16 kg for women of normal weight and 5 to 9 kg for women with obesity.

### 2.2. Gestational Diabetes

Screening for gestational diabetes was based on a fasting blood glucose (FBG) in the first trimester for women with BMI ≥ 30 kg/m^2^, and a 75g oral glucose tolerance test (OGTT) between 24 and 28 weeks regardless of maternal BMI. A limited number of women with BMI 18.5–24.9 kg/m^2^ also had a first trimester FBG prior to booking. In addition to this routine practice, all participants were screened for glucose intolerance at 32 weeks by repeating 75g-OGTT. Gestational diabetes was defined according to the thresholds published by the International Association of Diabetes and Pregnancy Study Groups (IADPSG) for the second and third trimester [12]. The first line treatment was dietary intervention with a standard 1800 kilocalories daily meal plan divided into three meals and two snacks, and self-capillary blood glucose monitoring. The objective was to maintain fasting glucose level <90 mg/dL and post-prandial level <120 mg/dL. Patients who were unable to achieve the established goals by dietary control after two weeks were prescribed insulin treatment.

### 2.3. Neonatal Characteristics

Skinfold thickness was measured with a skinfold caliper (Harpenden, Baty, UK) within 72 h of delivery, in practice between the second and the third day of life. To ensure accuracy and reproducibility of the measurement, the pediatricians in both centers attended a dedicated training course. Measurement of skinfold thickness was done at four sites: triceps, biceps, suprailiac, and subscapular, according to the method described by Schmelzle [13]. If two measurements differed by more than 0.5 mm, at least one extra measurement was taken until two similar measurements were obtained. The average of the two closest measurements at each site was calculated and the sum of the values at the four sites was used as a proxy for neonatal fat mass.

### 2.4. Biological Assays

Cord blood was collected immediately after delivery to measure leptin. Leptin was measured by ELISA (leptin, Biovendor, Eurobio, Les Ulis, France), according to manufacturer’s instructions with inter-assay coefficient variation of 7.2%.

### 2.5. Statistical Analysis

Data were expressed as number (percent) for qualitative variables and mean (SD) for quantitative variables. We used Pearson’s Chi-squared tests or Fisher’s exact tests to compare qualitative variables between groups, and Student test or Wilcoxon rank sum tests for quantitative variables. We used Lin’s concordance correlation coefficient (CCC) to evaluate the agreement between declared pre gestational maternal weight and weight measured at inclusion [14].

In order to identify pregnancy weight gain profiles, we used a latent class linear mixed model [15]. In this model, we introduced the interaction between gestational age and maternal obesity as fixed effect, and gestational age and the subject as random effects. The time function was assumed to be linear.

In order to assess the association between pregnancy weight gain and neonatal weight and fat mass estimated by skinfolds and leptin, we used two linear models. in which dependent variables were birthweight, skinfolds, and cord leptin.

To evaluate multicollinearity, the variance inflation factor (VIF) was calculated for each covariate included in multivariate analyses. VIF < 1.5 is an indicator of the absence of multicollinearity.

Model I was fitted without interactions allowing to estimate the apparent adjusted effect on dependent variables of (a) each kg of GWG, (b) the presence of gestational diabetes, (c) pre-pregnancy obesity, and (d) neonatal sex.

In model II, interactions between pregnancy weight gain and maternal obesity, gestational diabetes and sex of the neonate were added. This enabled us to estimate the difference in the effect of GWG on dependent variables between (a) obese and non-obese women, (b) women with or without gestational diabetes, and (c) women giving birth to a boy or to a girl.

We also conducted analyses using the same models by estimating the effect of 1 kg GWG in reference to the weight gain recommended by the IOM depending on the BMI category (11.5 to 16 kg for normal weight women and 5 to 9 kg for obese women). So, we used the class centre of recommended weight gain as reference value, i.e., 13.75 kg and 7 kg for normal weight women and obese women, respectively. The relative variation of weight gain was defined as GWG minus reference value.

All analyses were performed at a two-sided α level of 5%, using R software, version 3.5.1 (R Foundation for Statistical Computing, Vienna, Austria. URL https://www.R-project.org/. including lcmm package [15].

## 3. Results

**Figure 1:**
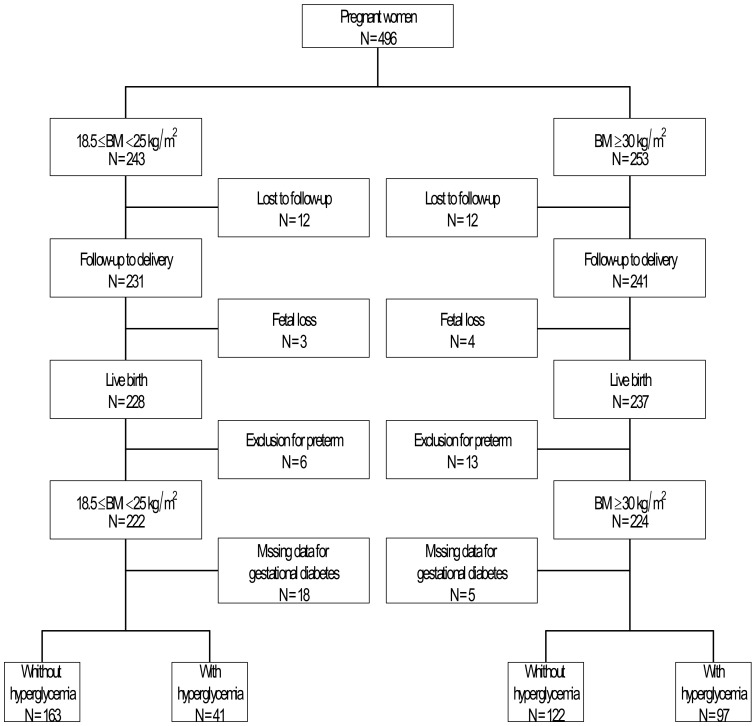
Flowchart of the study. BMI: Body mass Index.

Of 496 pregnant women enrolled, 24 were lost to follow up during pregnancy, seven had a fetal loss, 19 had a preterm delivery, and data on glucose metabolism were missing in 23, resulting in a database of 423 pregnancies with a term live born and a complete follow-up (Figure 1). Two hundred and four neonates were born to women with BMI 18.5–24.9 kg/m^2^ and 219 to women with BMI ≥ 30 kg/m^2^.

### 3.1. Characteristics of Maternal GWG

The self-reported pre-pregnancy weight was in agreement with the weight measured at the first prenatal visit, with a concordance coefficient correlation of 0.96 (CI 95%, 0.95 to 0.97).

Table 1 shows maternal and neonatal characteristics. In obese women, total and relative GWG were significantly lower than in women with BMI 18.5–24.9 kg/m^2^, but the rate of GWG exceeding IOM recommendations was twice greater (50%) than in non-obese women.

Within the entire cohort, using a latent class linear mixed model, two GWG profiles were identified: a group with high GWG (*N* = 387), characterized by a mean GWG of 11.40 kg and a group with low GWG (*N* = 36), characterized by a mean GWG of 2.95 kg. The two groups were significantly different by the average pre-pregnancy BMI. In the first group, mean pre-pregnancy BMI was 26.8 kg/m^2^ (min 18.5/max 40.5). In the second group, mean pre-pregnancy BMI was 43.1 kg/m^2^ and all women in this group had a pre-pregnancy BMI > 35 kg/m^2^ (min 35.5/max 52.7). The mean birthweight was not significantly different between the high and the low GWG group. However, skinfolds were significantly thicker in neonates born to mothers in the lower GWG group than in those born to mothers in the higher GWG groups (20.1 mm versus 18.6 mm, *p* = 0.02).

### 3.2. Contribution of Total GWG to Birthweight, Skinfolds, and Leptin

The reference (intercept) was a theoretical boy-neonate born to a normal weight woman, without diabetes and with zero-gestational weight gain. Because maternal obesity is associated with gestational diabetes, multicollinearity was assessed between covariates included in multivariate analyses. We did not detect collinearity between the covariates (Table S1).

According to model I (Table 2), there was a significant association between total GWG and birthweight and newborn skinfolds thickness. On average one-kilogram total GWG increased birthweight by 9.9 g and skinfolds thickness by 0.08 mm There was also a significant association between gestational diabetes and newborn skinfolds thickness (+0.91 mm). There was a greater impact of maternal obesity than total GWG or diabetes on the increase in skinfolds thickness (+1.63 vs. 0.08 and 0.91 mm, respectively) and cord leptin (+2.88 vs. 0.12 and 0.85 ng/mL, respectively).

Model II was designed to assess the interactions between GWG, maternal obesity, gestational diabetes, and sex of the neonate regarding their effect on birthweight, skinfolds, and leptin. According to this model (Table 2), the contribution of total GWG on birthweight and skinfolds thickness was not modified by maternal obesity, gestational diabetes or sex of the newborn. On the other hand, the contribution of total GWG on the average cord leptin level was significantly greater in case of maternal diabetes with a 0.37 ng/mL increase. The effect of total GWG on the dependent variables was not affected by the sex of the neonate.

To consider a differential effect of one-kilogram total GWG in normal weight and obese women, we also conducted analyses using the same models by estimating the effect of 1 kg gestational weight gain in reference to the class centre of weight gain recommended by the IOM depending on the maternal BMI category. The mean [SD] relative variation of weight gain (defined as GWG minus class centre value) was −0.47 kg (4.26) for normal weight women and 1.18 kg (7.52) for obese women. The results of these analyses were similar to those found by applying the GWG per kg in the linear models (Table S2).

### 3.3. Contribution of Trimester-Specific GWG on Birthweight, Skinfolds, and Leptin

According to model I, there was no association between weight gain in the first and second trimesters, and either of birthweight, skinfolds thickness, or cord leptin level (Tables S3 and S4). One-kilogram third trimester weight gain, however, was associated with a 13.4 g increase in birthweight (marginally significant with *p* = 0.053), a 0.17 mm increase in skinfolds thickness (*p* = 0.002) and a 0.64 ng/mL increase in cord leptin (*p* < 0.001) (Table 3). Birthweight was not affected by gestational diabetes and maternal obesity at any time of pregnancy. They were, however, associated with higher neonatal skinfolds thickness and cord leptin level, with a greater effect of obesity.

According to model II, the effect of maternal weight gain every trimester during pregnancy on the increase in birthweight was not modified by maternal obesity (Table 3, Tables S3 and S4). The effect of third trimester weight gain on birthweight was increased by the presence of gestational diabetes (+32.2 g for one-kilogram weight gain, *p* = 0.023). Neither maternal obesity or gestational diabetes modified the effect of GWG on the increase in skinfold thickness, at any trimester of pregnancy. In contrast, the effect of first and third trimester weight gain on the increase in cord leptin level was significantly higher in newborns whose mothers had gestational diabetes (respectively for one-kilogram weight gain, +0.47 ng/mL, *p* = 0.038 and +0.89 ng/mL, *p* = 0.005). No interaction was found between third trimester weight gain and the sex of the neonate.

## 4. Discussion

### 4.1. Principal Findings

Our study found an association between GWG, birthweight, and neonatal fat mass, after adjustment for maternal obesity and gestational diabetes, and without finding significant collinearity between these factors in the multivariate model. This association seemed mainly due to third trimester weight gain. We also confirm that gestational diabetes is associated with neonatal fat mass and that it enhances the effect of third trimester weight gain on birthweight and fat mass. Our main result however, is that obesity has the greater impact on neonatal fat mass and that the association between maternal obesity and increased neonatal fat mass cannot be explained by a specific pattern of weight gain. Moreover, the effect of weight gain does not differ according to the sex of the newborn. Because neonatal fat mass is associated with increased cardio-metabolic risk in childhood and adolescence as well as in adulthood [16], our findings suggest that pre pregnancy maternal nutritional status should be optimized.

### 4.2. Strengths and Limitations

The strength of this study is the fact that it is based on a well-characterized prospective cohort of mother–infant dyads designed to explore neonatal body fat mass in addition to birthweight. All women had access to the same health care, and maternal body weight was measured monthly since the first trimester onwards. Self-reported pre-pregnancy weight was highly correlated with weight recorded at the first prenatal visit suggesting our estimate of GWG was reliable. The main weakness of our study is its observational design, preventing interpreting association of variables as a causal relationship, and not taking into account some potential unrecognized confounding factors.

In addition, we are aware that some associations may have appeared significant by mere chance. Another potential limitation results from using skinfold measurements as a proxy for neonatal fat mass. In neonates, skinfold thickness is an indirect measurement of adiposity, but it correlates with fat mass values determined by dual-energy X-ray absorptiometry (DXA), a validated method for determining body fat [13]. Fetal leptin level is believed to correlate with fetal fat mass and to be independent from maternal and placental contributions [17]. All patients received the general pregnancy dietary recommendations but no specific intervention for weight management during pregnancy was proposed. Nevertheless, we cannot formally exclude that women included in the study have changed their lifestyle because of enrolment.

In contrast to many previous studies, we used GWG as a continuous variable instead of categories such as “below, within, or above the IOM recommendations”. Notwithstanding that these recommendations may be contentious in women with severe obesity [18,19], our choice was guided by the fact our perspective was more explanatory than pragmatic. This choice enabled us to detect minor associations between weight gain and the dependent variables we studied, but we reckon our results cannot be interpreted in terms of practical recommendations.

### 4.3. GWG, Birthweight, and Neonatal Fat Mass

Our findings of GWG being associated with neonatal weight is in accordance with previously published data. We found like others, that GWG is positively associated with birthweight and neonatal fat mass [3,7,8,20]. Although maternal GWG can influence fetal growth, it has been underlined recently that the timing of weight gain during gestation should be considered, because the effect of weight gain is different depending on the time of pregnancy [21]. There are controversies regarding the timing of weight gain during pregnancy and the effect on birthweight and few studies have explored neonatal fat mass as a function of the timing of pregnancy weight gain. In line with other studies, we found a strong effect of late GWG on fetal growth. Indeed, Karachaliou et al. [22] reported that greater second- and third-trimester rate of GWG was associated with increased birthweight Z-score and risk of LGA neonates. Gaillard et al. [23] observed that higher weight gain in each trimester was associated with a greater risk of delivering a LGA infant, but the strongest effects of weight gain were during the second and third trimester. Ruchat et al. [24] showed that excessive GWG in mid/late but not in early pregnancy, was associated with increased birthweight and that the excess of growth was partly related to an increase in neonatal adipose tissue. Indeed, we also found that third trimester weight gain was associated with higher skinfold thickness and cord leptin. In contrast, Broskey et al. [21] showed that regardless of maternal BMI, excessive GWG before 24 weeks of gestation was associated with an increased risk of LGA infants. Two other studies showed that when mothers had excessive weight gain in the first half of pregnancy, neonates were more likely to have significantly higher birthweight and body fat [25,26]. The difference in the contribution of GWG at different stages of pregnancy on birthweight and neonatal fat mass reported in these studies might be explained by the various methods used to estimate weight gain, whether or not diabetes is taken into account, and the ethnic and socio-economic characteristics of the populations studied.

### 4.4. Interaction between Diabetes and GWG

We found a positive interaction between both gestational diabetes and weight gain in the third trimester, and fetal growth despite the active screening and treatment of gestational diabetes. These results are in line with recent observations by Aiken et al. [27] in a cohort of women with a diagnosis of gestational diabetes. In this study, the authors showed that excessive weight gain after the diagnosis of diabetes (>28 weeks) increased the risk of having a LGA newborn, despite the treatment of diabetes. This underlines the fact that in case of maternal diabetes, placental transfer of energy substrates is enhanced by the modified expression of transporters for glucose, lipids, and amino acids and that this is probably not reversed by the treatment of diabetes [9]. Furthermore, the fetus of a diabetic mother would have developed pancreatic hyperplasia by the time the diagnosis of diabetes is made between 24 to 28 weeks or even later, and already has inappropriate insulin secretion that persists along with pregnancy, despite treatment and good glycemic control. As a result, there is an inappropriate secretory response to any glucose load, or other insulin secretagogues such as amino acids and fatty acids that leads to excessive fetal growth [28].

### 4.5. The Specific Effect of Maternal Obesity on Fat Mass

In the studied cohort, we previously showed that regardless of gestational diabetes, maternal obesity was not associated with birthweight, but it was associated with fat mass as estimated by skinfold thickness and leptin in girls only [10]. We have confirmed, in this study, that maternal obesity independently increased neonatal fat mass and we showed that it had the greater impact on fat mass as compared to GWG and diabetes. Additionally, we have showed in a subgroup of women with a BMI > 35 kg/m^2^ and low GWG (<3 kg) that skinfold thickness was higher than in the group including women with normal weight and obese women with mean weight gain of 11.4 kg. The specific effect of maternal obesity on neonatal fat mass could explain the findings that the associations of excessive GWG with being LGA was attenuated when maternal obesity was taken into account [23]. Furthermore, in a prospective cohort, pre-pregnancy BMI was the largest contributor to newborn fat mass whereas excessive GWG above IOM recommendations in obese women had no effect on newborn fat mass [29].

Thus, the effect of GWG on neonatal fat mass was lower than that of maternal obesity, suggesting the association between maternal obesity and neonatal fat mass is mediated by a mechanism independent from maternal weight gain.

### 4.6. Hypothesized Mechanisms

There is a major gap in our understanding of the regulation of fetal fat accretion, which may vary according to the stage of pregnancy and is affected by maternal diabetes and maternal obesity. Throughout pregnancy, maternal metabolism changes to enable fetal growth. For example, insulin sensitivity decreases, which favors the transfer of nutrients to the fetus [16]. Maternal diabetes and obesity alter the intrauterine environment and act on different placental molecular targets [30], promoting the transport of a number of nutrients [9]. Excessive GWG also alters the expression of genes involved in regulating placental nutrient transport [31]. Some authors consider that the metabolic situation of obese women leads to changes in placental gene expression and function very early in pregnancy, which may explain the predominant effect of maternal obesity on fetal fat mass [16].

Furthermore, altered maternal and intrauterine environment due to diabetes and obesity can modify fetal regulatory gene activity and expression through epigenetic mechanisms that pave the way for fetal programming [32,33]. Before birth, it represents an adaptive change of the fetus to an adverse environment that, after delivery, results in altered metabolic responses to the postnatal environment.

These epigenetic changes may affect the fetal hormonal milieu, as for example the increase in leptin synthesis and concentrations [34]. This may explain our observation that third trimester GWG was associated with an increase in leptin level, but not skinfold thickness, in cases of gestational diabetes. Leptin dysregulation alters the development and function of organs such as the hypothalamus, which contributes to the regulation of satiety, increasing the likelihood of developing diseases in adulthood [35].

The mechanisms underlying the sexual difference in the accumulation of fetal fat mass in offspring from obese women remain unclear. Being the active interface between the mother and the fetus, the placenta appears to be a relevant target to better understand the molecular link between maternal obesity, insulin-resistance, and fetal growth. Recently, different studies in women with high BMI showed that placental adaptations to maternal environment, including placental biometry or histopathology, differ according to fetal sex, with significant changes so far reported in girls only [36,37]. Nevertheless, Brass et al. [38] showed that placental uptake of oleic acid was suppressed and that the expression of the placental transporter CD36 was lower in male but not in female newborn of obese women. Recently, it was shown that miRNA expression in amniotic fluid and fetal hepatocytes is dependent on sex in offspring of women with GDM or obesity, although the mechanisms contributing to this phenomenon remain unknown [39].

In addition to showing an increase in fat mass in neonates from obese women, we did not show an increase in birth weight. This suggests that lean mass may be decreased although whole body composition was not evaluated in our study. In utero exposure to maternal diabetes has been linked to a reduction in lean body mass, with an effect on lean mass until early childhood and adolescence [40]. In animal models, prenatal exposure to nutrient excess has been linked to impaired myogenesis [41]. Paucity of muscle mass may be a determinant for diabetes in the future, as muscle is an important insulin target tissue and decreased muscle mass is linked to insulin resistance.

However, the mechanisms involved in abnormal maternal metabolic environment and subsequent changes in key organs involved in programming are far from being fully understood. Understanding the complex interactions between GWG at any time of pregnancy, taking into account maternal pre-pregnancy BMI, diabetes, and other maternal genetic and environmental factors requires further studies. This is critical to decipher the impact of these maternal conditions on offspring outcomes.

## 5. Conclusions

Our results suggest that GWG independently affects birthweight and neonatal fat mass, mainly during third trimester and this effect is enhanced by gestational diabetes, whereas maternal obesity is the main contributor to neonatal body fat mass. Dietary interventions, exercise, and the treatment of gestational diabetes may limit GWG and macrosomia. These interventions should be promoted although they are not likely to be entirely sufficient to control the increase in neonatal body fat mass associated with maternal obesity. Interventions to reduce or prevent maternal obesity before conception may probably be the best way to improve maternal health and limit adverse consequences for the offspring.

## Figures and Tables

**Table 1:** Maternal and neonatal characteristics according to maternal BMI. Data are expressed as mean (SD) or *N* (%). * according to the Institute of Medicine (IOM) recommendations. BMI: Body Mass Index; NS: not significant.

	18.5 ≤ BMI < 25 kg/m^2^ *N* = 204	BMI ≥ 30 kg/m^2^ *N* = 219	*P*-Value
Maternal characteristics			
Age (years)	31.0 (4.1)	30.7 (4.7)	NS
Primiparity *n* (%)	72 (35.0)	87 (40.0)	NS
Gestational age at inclusion (weeks)	15.3 (2.1)	15.2 (2.3)	NS
BMI before pregnancy (kg/m^2^)	21.3 (1.7)	34.7 (4.6)	<0.0001
Ethnicity *n* (%)			<0.0001
White European	141 (69.5)	74 (34.1)	
Northern Africa	17 (8.4)	50 (23.0)	
Sub Saharan Africa	28 (13.8)	86 (39.6)	
Other	17 (8.4)	7 (3.2)	
Gestational diabetes *n* (%)	41 (20.0)	97 (44.0)	<0.0001
Total gestational weight gain (kg)	13.3 (4.3)	8.2 (7.5)	<0.0001
Relative weight gain (%)	22.9 (7.5)	9.1 (7.9)	<0.0001
1st trimester weight gain	5.4 (3.1)	3.0 (5.0)	<0.0001
2nd trimester weight gain	3.6 (2.6)	2.4 (2.7)	
3rd trimester weight gain	4.4 (2.5)	2.9 (3.8)	
Within recommended range *n* (%) *	91 (48.0)	48 (23.0)	<0.0001
More than recommended *n* (%)	49 (26.0)	103 (50.0)	
Less than recommended *n* (%)	51 (27.0)	57 (27.0)	
Neonatal characteristics			
Gestational age (weeks)	39.7 (1.1)	39.6 (1.1)	NS
Boys *n* (%)	106 (52)	99 (45)	NS
Birthweight (g)			
Boys	3466 (427)	3397 (463)	NS
Girls	3307 (410)	3396 (405)	NS
Sum of skinfolds (mm)			
Boys	18.2 (3.5)	18.9 (4.3)	NS
Girls	17.7 (3.0)	19.9 (3.6)	<0.0001
Cord blood leptin (ng/mL)			
Boys	8.9 (8.2)	9.0 (6.5)	NS
Girls	10.6 (8.1)	15.4 (11.2)	0.001

**Table 2:** Contribution of total gestational weight gain on birth weight, skinfold thickness, and cord leptin level.

	Birthweight (g)			Skinfold Thickness (mm)			Cord Leptin (ng/mL)		
Model I	Estimate	CI 95%	*p*	Estimate	CI 95%	*p*	Estimate	CI 95%	*p*
(Intercept)	3298.3	3180.2–3416.5	<0.001	16.54	15.52–17.56	<0.001	6.14	3.42–8.87	<0.001
Gestational weight gain (per kg)	9.9	3.1–16.7	0.004	0.08	0.02–0.14	0.009	0.12	−0.04 to 0.27	NS
Gestational diabetes	26.9	−66.4 to 120.2	NS	0.91	0.11–1.72	0.027	0.95	−1.18 to 3.07	NS
Obesity	36.2	−55.3 to 127.8	NS	1.63	0.84–2.42	<0.001	2.88	0.77–5.00	0.008
Newborn sex (girl)	−79.9	−163.0 to 3.2	0.059	0.33	−0.38 to 1.05	NS	4.00	2.08–5.92	<0.001
Model II									
(Intercept)	3205.855	2991.2–3420.5	<0.001	16.19	14.35–18.03	<0.001	6.40	1.46–11.33	0.011
Gestational weight gain (per kg)	17.174	1.4–32.9	0.032	0.10	−0.03 to 0.23	NS	0.09	−0.26 to 0.45	NS
Gestational diabetes	86.766	−76.0 to 249.5	NS	0.57	−0.82 to 1.95	NS	−2.94	−6.65 to 0.77	NS
Obesity	102.848	−117.5 to 323.2	NS	2.39	0.51–4.26	0.013	4.67	−0.36 to 9.70	NS
Newborn sex (girl)	−77.563	−235.9 to 80.8	NS	0.15	−1.20 to 1.51	NS	5.22	1.64–8.79	0.004
Gestational weight gain: gestational diabetes	−6.321	−19.4 to 6.7	NS	0.03	−0.08 to 0.14	NS	0.37	0.08–0.66	0.013
Gestational weight gain: obesity	−5.304	−21.7 to 11.1	NS	−0.06	−0.20 to 0.08	NS	−0.15	−0.52 to 0.22	NS
Gestational weight gain: newborn sex (girl)	−0.007	−12.6 to 12.6	NS	0.02	−0.09 to 0.12	NS	−0.12	−0.40 to 0.16	NS

Model I (multivariate analysis) was fitted to estimate the independent effect of (a) each kg of gestational weight gain (GWG), (b) the presence of gestational diabetes, (c) pre-pregnancy obesity, and (d) neonatal sex on birthweight, skin fold thickness, and cord leptin. In model II, interactions between gestational weight gain and maternal obesity, gestational diabetes, and sex of the neonate were added, in order to estimate the difference in the effect of GWG on dependent variables between (a) obese and non-obese women, (b) women with or without gestational diabetes, and (c) women giving birth to a boy or to a girl. Gestational weight gain was considered as a continuous variable. NS: not significant.

**Table 3:** Contribution of third trimester gestational weight gain on birth weight, skinfold thickness, and cord leptin level.

	Birthweight (g)			Skinfold Thickness (mm)			Cord Leptin (ng/mL)		
Model I	Estimate	CI 95%	*p*	Estimate	CI 95%	*p*	Estimate	CI 95%	*p*
(Intercept)	3375.8	3277.4–3474.3	<0.001	16.83	16.05–17.61	<0.001	4.33	2.10–6.56	<0.001
3rd trimester weight gain (per kg)	13.4	−0.19 to 27.1	0.053	0.17	0.06–0.28	0.002	0.64	0.33–0.96	<0.001
Gestational diabetes	22.1	−75.5 to 119.7	NS	0.78	−0.00 to 1.56	0.051	2.09	−0.03 to 4.21	0.053
Obesity	9.2	−79.9 to 98.4	NS	1.42	0.71–2.13	<0.001	3.50	1.52–5.47	0.001
Newborn sex (girl)	−86.7	−172.0 to −1.4	0.046	0.31	−0.37 to 0.99	NS	3.90	2.02–5.78	<0.001
Model II									
(Intercept)	3389.3	3242.5–3536.1	<0.001	17.17	16.00–18.33	<0.001	2.52	−0.76 to 5.80	0.132
3rd trimester weight gain (per kg)	10.3	−19.4 to 40.0	NS	0.08	−0.15 to 0.32	NS	1.09	0.44–1.75	0.001
Gestational diabetes	−84.1	−217.1 to 49.0	NS	0.23	−0.83 to 1.30	NS	−0.90	−3.82 to 2.03	NS
Obesity	76.7	−72.8 to 226.3	NS	1.68	0.49–2.87	0.006	6.91	3.59–10.23	0.000
Newborn sex (girl)	−94.3	−221.4 to 32.7	NS	−0.13	−1.15 to 0.89	NS	5.59	2.77–8.41	0.000
3rd trimester weight gain: gestational diabetes	32.2	4.4–60.0	0.023	0.15	−0.07 to 0.37	NS	0.89	0.27–1.51	0.005
3rd trimester weight gain: obesity	−15.0	−44.7 to 14.8	NS	−0.05	−0.29 to 0.18	NS	−0.82	−1.48 to −0.15	0.016
3rd trimester weight gain: newborn sex (girl)	−0.1	−26.1 to 25.8	NS	0.11	−0.10 to 0.31	NS	−0.52	−1.10 to 0.06	NS

Model I (multivariate analysis) was fitted to estimate the independent effect of (a) each kg of third trimester weight gain, (b) the presence of gestational diabetes, (c) pre-pregnancy obesity, and (d) neonatal sex on birthweight, skin fold thickness, and cord leptin. In model II, interactions between pregnancy weight gain and maternal obesity, gestational diabetes, and sex of the neonate were added, in order to estimate the difference in the effect of third trimester weight gain on dependent variables between (a) obese and non-obese women, (b) women with or without gestational diabetes, and (c) women giving birth to a boy or to a girl. Gestational weight gain was considered as a continuous variable. NS: not significant.

## Supplementary Materials

The following are available online at https://www.mdpi.com/2072-6643/12/11/3434/s1, Table S1: Evaluation of the multicollinearity between the variables included in the multivariate analysis. Table S2: Contribution of 1kg weight gain relative to the class centre of recommended GWG by the IOM on birth weight, skinfold thickness and cord leptin level. Table S3: Contribution of 1st trimester gestational weight gain on birth weight, skinfold thickness and cord leptin level. Table S4: Contribution of 2nd trimester gestational weight gain on birth weight, skinfold thickness and cord leptin level.
