# Supplementary material for: Relative Contribution of Gestational Weight Gain, Gestational Diabetes, and Maternal Obesity to Neonatal Fat Mass
Source: Nutrients. 2020 Nov 9;12(11):3434. doi: 10.3390/nu12113434 (PMC7698189; doi:10.3390/nu12113434)
Supplement: Supplementary file 1 [file nutrients-12-03434-s001.pdf]

Variable	VIF
Gestational weight gain (kg)	1.20
Maternal obesity	1.22
Newborn sex	1.01
Gestational diabetes	1.09

Supplemental table 1: Evaluation of the multicollinearity between the variables included in the multivariate analysis. VIF: variance inflation factor.

Appendix A

	Birthweight (g)			Skinfold thickness (mm)			Cord leptin (ng/ml)		
**Model I**	**Estimate**	**CI 95%**	**p**	**Estimate**	**CI 95%**	**p**	**Estimate**	**CI 95%**	**p**
**(Intercept)**	3434.7	3360.7-3509.0	< 0.001	17.61	16.97 - 18.24	< 0.001	7.74	6.03 - 9.46	< 0.001
**Relative variation in gestational weight gain (per kg)**	9.9	3.1-16.7	0.004	0.08	0.02 - 0.14	0.009	0.12	-0.04 - 0.27	NS
**Gestational diabetes**	26.9	-66.4-120.2	NS	0.91	0.11 - 1.72	0.027	0.95	-1.18 - 3.07	NS
**Obesity**	-30.7	-117.7-56.3	NS	1.11	0.36 - 1.86	0.004	2.10	0.09 - 4.11	0.041
**Newborn sex (girl)**	-79.9	-163.0-3.2	0.059	0.33	-0.38 - 1.05	NS	4.00	2.08 - 5.92	< 0.001
**Model II**									
**(Intercept)**	3439.2	3364.4-3514.0	< 0.001	17.65	17.01 - 18.29	< 0.001	7.79	6.08 - 9.50	< 0.001
**Gestational weight gain (per kg)**	12. 8	-3.1-28.7	NS	0.06	-0.07 - 0.20	NS	0.00	-0.36 - 0.36	NS
**Gestational diabetes**	23.5	-70.8-117.8	NS	0.84	0.03 - 1.65	0.043	0.59	-1.54 - 2.73	NS
**Obesity**	-33.5	-122.2-55.2	NS	1.17	0.41 – 1.93	0.003	2.68	0.64 - 4.71	0.01
**Newborn sex (girl)**	-82.1	-165.4-1.2	NS	0.30	-0.42 - 1.02	NS	3.99	2.09 - 5.90	< 0.001
**Gestational weight gain : gestational diabetes**	-1,7	-15.7-12.2	NS	0.05	-0.06 - 0.17	NS	0.44	0.13 - 0.76	0.005
**Gestational weight gain : obesity**	-7.3	-23.8-9.1	NS	-0.08	-0.22 - 0.06	NS	-0.18	-0.55 - 0.19	NS
**Gestational weight gain : newborn sex (girl)**	7.0	-6.6-20.6	NS	0.09	-0.02 - 0.21	NS	0.05	-0.25 - 0.36	NS

Supplemental table 2. Contribution of 1kg weight gain relative to the class centre of recommended GWG by the IOM on birth weight, skinfold thickness and cord leptin level. Model I (multivariate analysis) was fitted to estimate the independent effect of (a) each kg of GWG, (b) the presence of gestational diabetes, (c) pre-pregnancy obesity, and (d) neonatal sex on birthweight, skin fold thickness and cord leptin. In model II, interactions between gestational weight gain and maternal obesity, gestational diabetes and sex of the neonate were added, in order to estimate the theoretical difference in the effect of GWG on dependent variables between (a) obese and non-obese women, (b) women with or without gestational diabetes, and (c) women giving birth to a boy or to a girl. Gestational weight gain was considered as a continuous variable. NS: not significant.

	Birthweight (g)			Skinfold thickness (mm)			Cord leptin (ng/ml)		
**Model I**	**Estimate**	**CI 95%**	**p**	**Estimate**	**CI 95%**	**p**	**Estimate**	**CI 95%**	**p**
**(Intercept)**	3388.7	3297.8-3479.5	< 0.001	17.39	16.59 - 18.19	< 0.001	8.08	6.03 - 10.13	< 0.001
**1st trimester weight gain (per kg)**	8.9	-1.0 - 18.8	NS	0.06	-0.03 - 0.14	NS	-0.07	-0.29 - 0.16	NS
**Gestational diabetes**	23.0	-68.0 - 114.1	NS	0.98	0.18 - 1.79	0.017	0.55	-1.47 - 2.56	NS
**Obesity**	22.8	-66.6 - 112.2	NS	1.33	0.55 - 2.12	0.001	2.33	0.32 - 4.34	0.024
**Newborn sex (girl)**	-84.1	-166.8 - -1.5	0.046	0.21	-0.52 - 0.94	NS	4.09	2.23 - 5.94	< 0.001
**Model II**									
**(Intercept)**	3373.277	3239.0 - 3507.6	< 0.001	17.06	15.85 - 18.26	< 0.001	10.70	7.68 - 13.73	< 0.001
**1st trimester weight gain (per kg)**	13.275	-8.9 - 35.4	NS	0.12	-0.08 - 0.31	NS	-0.59	-1.09 - -0.10	0.020
**Gestational diabetes**	126.116	5.1 - 247.1	0.041	1.03	-0.05 - 2.11	NS	-1.20	-3.92 - 1.52	NS
**Obesity**	-18.671	-160.4 - 123.1	NS	1.78	0.52 - 3.04	0.006	0.48	-2.75 - 3.70	NS
**Newborn sex (girl)**	-89.264	-204.0 - 25.5	NS	0.07	-0.95 - 1.09	NS	3.93	1.34 - 6.51	0.003
**1st trimester weight gain : gestational diabetes**	-25.249	-44.9 - 5.6	0.012	-0.02	-0.19 - 0.16	NS	0.47	0.03 - 0.90	0.038
**1st trimester weight gain : obesity**	10.729	-12.3 - 33.8	NS	-0.09	-0.29 - 0.11	NS	0.36	-0.16 - 0.88	NS
**1st trimester weight gain : newborn sex (girl)**	1.816	-17.3 - 21.0	NS	0.03	-0.13 - 0.20	NS	0.03	-0.39 - 0.46	NS

Supplemental table 3. Contribution of 1st trimester gestational weight gain on birth weight, skinfold thickness and cord leptin level. Model I (multivariate analysis) was fitted to estimate the independent effect of (a) each kg of 1st trimester weight gain, (b) the presence of gestational diabetes, (c) pre-pregnancy obesity, and (d) neonatal sex on birthweight, skin fold thickness and cord leptin. In model II, interactions between gestational weight gain and maternal obesity, gestational diabetes and sex of the neonate were added, in order to estimate the difference in the effect of 1st trimester weight gain on dependent variables between (a) obese and non-obese women, (b) women with or without gestational diabetes, and (c) women giving birth to a boy or to a girl. Gestational weight gain was considered as a continuous variable. NS: not significant.

	Birthweight (g)			Skinfold thickness (mm)			Cord leptin (ng/ml)		
**Model I**	**Estimate**	**CI 95%**	**p**	**Estimate**	**CI 95%**	**p**	**Estimate**	**CI 95%**	**p**
**(Intercept)**	3400.331	3304.6 – 3496.0	< 0.001	17.30	16.49 - 18.11	< 0.001	7.20	5.09 - 9.31	< 0.001
**2nd trimester weight gain (per kg)**	11.517	-4.5 – 27.5	NS	0.11	-0.03 - 0.25	NS	0.05	-0.31 - 0.40	NS
**Gestational diabetes**	22.278	-72.0 – 116.5	NS	0.92	0.13 - 1.71	0.023	0.98	-1.07 - 3.03	NS
**Obesity**	17.035	-72.0 – 106.1	NS	1.28	0.54 - 2.02	0.001	2.89	0.93 - 4.85	0.004
**Newborn sex (girl)**	-91.789	-176.7 - -6.9	0.034	0.17	-0.53 - 0.88	NS	4.00	2.12 - 5.87	< 0.001
**Model II**									
**(Intercept)**	3363.0	3238.9 - 3487.0	< 0.001	17.33	16.30 - 18.37	< 0.001	6.02	3.30 - 8.74	< 0.001
**2nd trimester weight gain (per kg)**	23.47	-6.0 - 52.9	NS	0.11	-0.14 - 0.36	NS	0.40	-0.25 - 1.05	NS
**Gestational diabetes**	42.3	-94.0 - 178.6	NS	0.32	-0.80 - 1.44	NS	-0.12	-3.09 - 2.84	NS
**Obesity**	36.18	-102.1 - 174.5	NS	0.93	-0.21 - 2.07	NS	4.49	1.49 – 7.50	0.003
**Newborn sex (girl)**	-60.8	-187.4 - 65.8	NS	0.95	-0.12 - 2.01	NS	5.34	2.56 - 8.12	0.000
**2nd trimester weight gain : gestational diabetes**	-8.4	-42.5 - 25.6	NS	0.23	-0.06 - 0.52	NS	0.32	-0.43 - 1.06	NS
**2nd trimester weight gain : obesity**	-6.3	-40.1 - 27.4	NS	0.14	-0.14 - 0.42	NS	-0.51	-1.24 - 0.23	NS
**2nd trimester weight gain : newborn sex (girl)**	-10.5	-42.0 - 20.9	NS	-0.25	-0.52 - 0.01	NS	-0.43	-1.12 - 0.26	NS

Supplemental table 4. Contribution of 2nd trimester gestational weight gain on birth weight, skinfold thickness and cord leptin level. Model I (multivariate analysis) was fitted to estimate the independent effect of (a) each kg of 2nd trimester weight gain, (b) the presence of gestational diabetes, (c) pre-pregnancy obesity, and (d) neonatal sex on birthweight, skin fold thickness and cord leptin. In model II, interactions between gestational weight gain and maternal obesity, gestational diabetes and sex of the neonate were added, in order to estimate the difference in the effect of 2nd trimester weight gain on dependent variables between (a) obese and non-obese women, (b) women with or without gestational diabetes, and (c) women giving birth to a boy or to a girl. Gestational weight gain was considered as a continuous variable. NS: not significant
